# Novel genomic variants influencing methotrexate delayed clearance in pediatric patients with acute lymphoblastic leukemia

**DOI:** 10.3389/fphar.2024.1480657

**Published:** 2024-11-14

**Authors:** Jung Yoon Choi, Hoshik Kwon, Hyery Kim, Kyung Taek Hong, Youngeun Ma, Kyung-Nam Koh, Sunmin Yun, Keon Hee Yoo, Sang Hoon Song, Ho Joon Im, Ju Han Kim, Hyoung Jin Kang

**Affiliations:** ^1^ Department of Pediatrics, Seoul National University Children’s Hospital, Seoul National University College of Medicine, Seoul, Republic of Korea; ^2^ Seoul National University Cancer Research Institute, Seoul, Republic of Korea; ^3^ Seoul National University Biomedical Informatics (SNUBI), Division of Biomedical Informatics, Seoul National University College of Medicine, Seoul, Republic of Korea; ^4^ Department of Pediatrics, Asan Medical Center Children’s Hospital, University of Ulsan College of Medicine, Seoul, Republic of Korea; ^5^ Department of Pediatrics, Seoul Metropolitan Children’s Hospital, Seoul, Republic of Korea; ^6^ Department of Pediatrics, Samsung Medical Center, Sungkyunkwan University School of Medicine, Seoul, Republic of Korea; ^7^ Department of Laboratory Medicine, Seoul National University College of Medicine, Seoul, Republic of Korea

**Keywords:** methotrexate, children, acute lymphoblastic leukemia, pediatric, pharmacogenomics, adverse reactions, delayed clearance

## Abstract

**Background:**

Methotrexate (MTX) is the primary drug used in the treatment of pediatric acute lymphoblastic leukemia (ALL). However, some patients exhibit delayed clearance of high-dose (HD) MTX, which induces severe nephrotoxicity, mucositis, hepatotoxicity, and neurotoxicity. We sought to identify relevant variants associated with delayed clearance of HD-MTX in pediatric patients with ALL.

**Methods:**

Whole-exome sequencing of germline DNA was performed in 51 Korean pediatric patients with ALL. A total of 341 HD-MTX infusion data points from 51 patients were analyzed. MTX levels and laboratory measurements reflecting toxicity outcomes were obtained. Correlations between peak serum MTX levels at 24 h and toxicity outcomes were assessed. Analyses were performed to identify variants affecting delayed MTX clearance.

**Results:**

The 24 h MTX level strongly correlated with the subsequent creatinine (Cr) level. Moreover, rs2229866 in *contactin 2* (*CNTN2*), rs200687372 in *myotubularin Related Protein 9* (*MTMR9*), rs777260512 in *polymerase iota* (*POLI*), rs16954698 in *polycystic kidney disease 1-like 2* (*PKD1L2*), rs117765468 in *NSE1 Homolog, SMC5-SMC6 Complex Component* (*NSMCE1*)*,* and rs1800956 in *endoglin* (*ENG*) were identified as candidate variants associated with delayed MTX clearance. In particular, *ENG* rs1800956 was significantly associated with delayed MTX clearance in all analyses and *PKD1L2* rs16954698 was replicated in an external dataset (phs000637.v1.p1) from the Database of Genotypes and Phenotypes (dbGaP).

**Conclusion:**

This is the first whole-exome sequencing-based analysis of delayed MTX clearance in pediatric patients with ALL. *ENG* rs1800956 and *PKD1L2* rs16954698 were found to be potentially influential variants associated with delayed MTX clearance. These findings provide insights into HD-MTX-induced nephrotoxicity and may contribute to reducing adverse reactions through treatment modification.

## 1 Introduction

HD-MTX plays an important role in the treatment of childhood and adult cancers, such as ALL, lymphoma, and osteosarcoma (Treon and Chabner, 1996). It has been used in consolidation therapy for ALL to overcome resistance and prevent central nervous system (CNS) relapse (Larsen et al., 2016). However, the delayed clearance of HD-MTX induces multiple side effects, including nephrotoxicity, mucositis, hepatotoxicity, and neurotoxicity. To prevent toxicity, proper hydration with alkalinization and leucovorin rescue are administered, while nonsteroidal anti-inflammatory drugs, penicillin, and aspirin-containing medications should be avoided in combination with MTX. Studies emphasized the need to monitor serum MTX levels, perform liver and renal function tests, and decide whether to increase leucovorin or use glucarpidase (carboxypeptidase G2) at the right time (Ramsey et al., 2018). In South Korea, to reduce HD-MTX toxicity, the infusion time was shortened from 24 h to 4 h compared with the COG AALL0232 protocol (Larsen et al., 2016). Despite appropriate efforts, severe renal toxicity occurs in some patients, requiring dialysis and sometimes leading to chronic renal disease. Therefore, many studies have aimed to elucidate the interindividual variability in the pharmacokinetics explained by pharmacogenomics of MTX (Song et al., 2021; Taylor et al., 2021). However, most previous studies were conducted based on candidate gene panel analyses with a few genes or mutations related to the folate pathway or transporter genes. Very few studies have conducted whole-genome or whole exome sequencing (WES).

Racial and ethnic disparities were observed in adverse drug events, and studies involving Asians were fewer compared to other races, such as white people, black people, and Hispanics (Baehr et al., 2015). Despite the growing need to consider racial factors when determining MTX toxicities or responses (Torres et al., 2022; Xu et al., 2022; Liu et al., 2024), no genomic research has been performed on the pharmacokinetics of MTX in childhood ALL in South Korea. In this study, we aimed to investigate the genomic variants related to delayed clearance of HD-MTX using WES in pediatric patients with ALL.

## 2 Materials and methods

### 2.1 Patients and clinical data

Clinical data and DNA were collected from 51 Korean pediatric patients with ALL treated with HD-MTX at three hospitals: Seoul National University Hospital (SNUH), Asan Medical Center (AMC), and Samsung Seoul Medical Center (SMC) between August 2012 and July 2019. HD-MTX was administered to patients with T-cell ALL, slow early responders in high-risk B-cell ALL, testis involvement, CNS-3 status, or >100,000/μL white blood cells at diagnosis. HD-MTX was included in two interim maintenance (IM) courses (four HD-MTX sessions every 2 weeks per the IM course) of the treatment protocol. Intrathecal methotrexate was administered every 4 weeks, and 6-mercaptopurine 25 mg/m^2^ was administered daily. Accordingly, patients were administered a maximum of eight doses of HD-MTX (5 g/m^2^/dose) as a 4-h infusion according to the treatment protocol. The clinical data of 341 HD-MTX infusions were collected from 51 patients.

To monitor MTX-induced toxicity, serum MTX levels and laboratory results as toxicity markers were collected until the level <0.1 μmol/L was confirmed twice in each MTX administration. Methotrexate level was measured by the homogenous enzyme immunoassay (ARK Diagnostics, Fremont, CA, USA), and limit of detection and quantitation was 0.02 and 0.04 μmol/L, respectively.The toxicity markers consisted of serial serum levels of Cr, blood urea nitrogen (BUN), alanine aminotransferase (ALT), aspartate aminotransferase (AST), and total bilirubin (TB). Cr, BUN, ALT, AST, and TB prefixed with “Baseline” were measured before HD-MTX administration (Table 1; Supplementary Table S1). Toxicity grade was defined according to the Common Terminology Criteria for Adverse Events (CTCAE) version 4.0 of the National Cancer Institute, and the estimated glomerular filtration rate (eGFR) was calculated using the Cr-based bedside Schwartz equation (Schwartz and Work, 2009).

**TABLE 1:** Comparison of the total 341 infusions and 51 infusions showing max fold Cr.

Characteristics	Total infusions (n = 341)		Infusions showing max fold Cr (n = 51)		P *
	Mean ± SD	N	Mean ± SD	N	
Sex (Male, %)		187 (54.9%)		28 (54.8%)	1
Age (year)	9.78 ± 4.42	341	9.72 ± 4.53	51	0.8862
Height (cm)	135.95 ± 25.87	339	135.96 ± 26.34	51	0.9989
Weight (kg)	36.04 ± 17.54	339	35.39 ± 16.52	51	0.8956
BSA (m^2^)	1.15 ± 0.38	339	1.14 ± 0.38	51	0.9241
BMI (kg/m^2^)	18.18 ± 3.39	339	17.94 ± 2.93	51	0.8374
Baseline Cr (mg/dL)	0.44 ± 0.44	341	0.38 ± 0.15	51	0.1253
Baseline eGFR	147.04 ± 39.19	339	160.75 ± 39.48	51	0.0132
MTX dosage (mg/m^2^)	4914.01 ± 260.88	339	4925.49 ± 218.93	51	0.7776
Numbers of infusions per dosing cycle					
1st		51 (15.0%)		16 (31.4%)	0.0992
2nd		48 (14.1%)		9 (17.6%)	
3rd		46 (13.5%)		7 (13.7%)	
4th		46 (13.5%)		3 (5.9%)	
5th		42 (12.3%)		5 (9.8%)	
6th		41 (12.0%)		6 (11.8%)	
7th		37 (10.9%)		3 (5.9%)	
8th		30 (8.8%)		2 (3.9%)	
MTX level (μmol/L)					
at 24h	2.69 ± 5.48	332	6.36 ± 9.79	50	0.0048
at 48h	0.56 ± 1.54	333	1.90 ± 3.62	49	0.0037
at 72h	0.24 ± 0.60	277	0.77 ± 1.32	46	0.0032
at 96h	0.17 ± 0.41	180	0.58 ± 0.78	34	0.0017
at 120h	0.19 ± 0.34	86	0.52 ± 0.51	24	0.0021
at 144h	0.25 ± 0.45	44	0.68 ± 0.62	14	0.0007
at 168h	0.25 ± 0.25	25	0.35 ± 0.26	16	0.0996
max Cr (mg/dL)	0.52 ± 0.51	341	0.65 ± 0.35	51	0.0007
min eGFR (mL/min/1.73 m^2^)	123.87 ± 30.85	339	100.88 ± 32.66	51	<0.0001
MTX DC ^†^		80 (23.5%)		25 (49.0%)	0.0002
CTCAE AKI					
grade 0		297 (87.1%)	24 (47.0%)		<0.0001
grade 1		30 (8.8%)	14 (27.5%)		
grade 2		11 (3.2%)	10 (19.6%)		
grade 3		2 (0.6%)	2 (3.9%)		
grade 4		1 (0.3%)	1 (2%)		

SD, standard deviation; BSA, body surface area; BMI, body mass index.

*Wilcoxon rank sum test for continuous variables and Chi-squared test for categorical variables.

^†^MTX, delayed clearance was defined as serum MTX, level (μmol/L) at 24 h ≥ 15 or 48 h ≥ 1.5 or 72 h ≥ 0.15 or 168 h ≥ 0.1.

This study was approved by the institutional review boards of SNUH, AMC, and SMC (IRB no. H-1702-109-833). Written informed consent was obtained from all patients and from the parents and/or legal guardians of patients aged <18 years.

### 2.2 WES and preprocessing

Germline DNA was extracted from the blood (n = 37), hair (n = 6), and bone marrow (n = 8). WES was performed, and data were obtained using a bioinformatics pipeline previously described (Park et al., 2019). Allele frequencies, consequences, and amino acid changes were annotated by *in silico* prediction using the Ensembl Variant Effects Predictor (McLaren et al., 2016). To assess the impact of variants, Sorts Intolerant From Tolerant (SIFT) (Kumar et al., 2009), Polymorphism Phenotyping v2 (PolyPhen-2) (Adzhubei et al., 2013), and Combined Annotation-Dependent Depletion (CADD) scores were utilized. Based on the authors’ recommendations and the literature (Castellana and Mazza, 2013; CADD, 2014), variants with a SIFT score of <0.05, a PolyPhen-2 score of >0.85, and a CADD score of >15 were classified as *deleterious*, *probably damaging*, and *deleteriousness*, respectively.

Tri-allelic variants, sex-chromosomal variants, and variants not reported in both the 1000 Genomes Project (1KGP) and the Genome Aggregation Database (gnomAD) were excluded from the statistical analysis (Genomes Project et al., 2015; Karczewski et al., 2020). From the initial list of variants, only nonsynonymous variants were included in the analysis. To remove false-positive variants, the reads of significant variants obtained from the statistical analysis were manually reviewed.

### 2.3 Correlation between serum MTX levels and toxicity markers and selection of representative infusions

**FIGURE 1:**
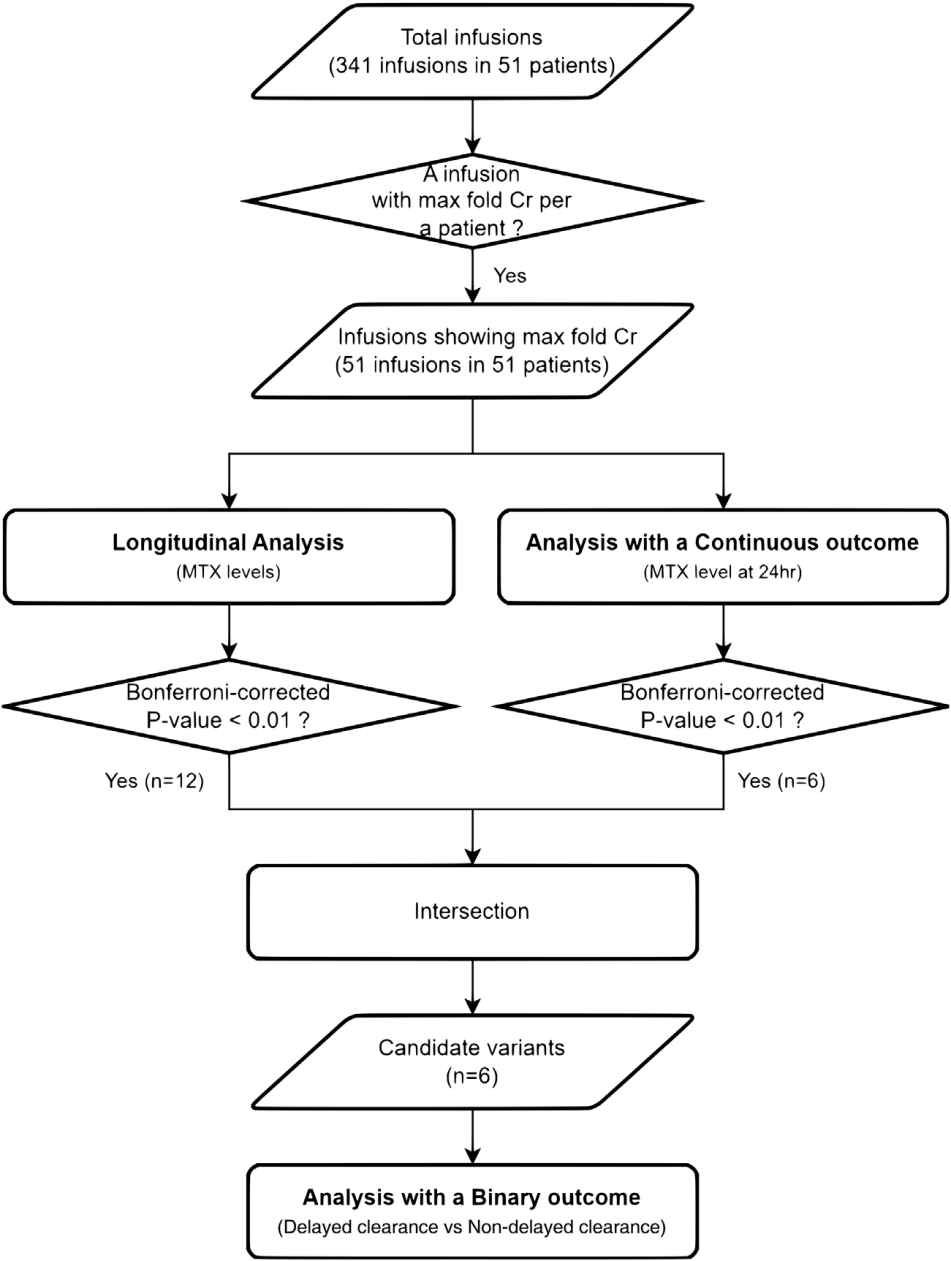
Flowchart of the analyses. From 51 Korean pediatric patients with ALL treated with HD-MTX, the infusion with the maximum fold change in creatinine was selected as the representative infusion for the statistical analyses.

Pearson’s correlation coefficient was used to assess the correlation between serum MTX levels and toxicity markers. The 24-h concentration of MTX was used as the peak level to estimate the correlation with toxicities, except for one patient without 24-h MTX level. For each infusion, the ratio of the baseline Cr to the post-infusion Cr values was defined as the fold change in Cr (fold Crs). An infusion with the maximum value among the fold Crs in one patient was defined as an infusion with the maximum-fold Cr (max fold Cr) in that patient. The infusion with the max fold Cr was selected as the patient’s representative infusion for statistical analysis (Figure 1).

### 2.4 Longitudinal analysis

To determine the difference in serum MTX levels by variants over time, a longitudinal analysis was performed using a linear mixed-effect model (LMM), assuming a dominant genetic model. Age, sex, and body mass index (BMI) were included as covariates in the LMM analysis. Genetic variants that satisfied the Bonferroni-corrected *p*-value <0.01 for variants over time were considered significant.

### 2.5 Analysis with a continuous outcome

Under the assumption of a dominant genetic model, multiple linear regression (MLR), adjusted for age, sex, and BMI, was used to evaluate the relationship between the 24-h MTX level and genetic variants. The threshold for the statistical significance of the variants was a Bonferroni-adjusted *p*-value of <0.01.

### 2.6 Analysis with a binary outcome

Six variants were derived by defining candidate variants as the intersection of the variants obtained in the continuous outcome and longitudinal analyses. Additional analysis was performed to identify significant associations (nominal *p* < 0.05) between candidate variants and delayed MTX clearance as a binary outcome (case–control) using Fisher’s exact test (FET). The case group was defined as serum MTX level (μmol/L) at 24 h ≥ 15, 48 h ≥ 1.5, 72 h ≥ 0.15, or 168 h ≥ 0.1, otherwise considered the controls (Santucci et al., 2010). Moreover, the toxicities were compared according to the genotypes of the variant with statistical significance in analyses.

### 2.7 Replication analysis using the external dataset

To validate the six candidate variants, an external dataset (phs000637.v1.p1) was utilized as a replication cohort from the Database of Genotypes and Phenotypes (dbGaP) (Mailman et al., 2007). This replication cohort consisted of 1,267 patients with B-precursor ALL and ≤21 years of age. The patients’ ancestry groups were White (n = 798; 63.0%), Hispanic (n = 266; 21.0%), Black (n = 57; 4.5%), Asian (n = 22, 1.7%), and other (n = 124; 9.8%). Patients received HD-MTX as part of protocol-directed therapy for ALL and corresponding serum MTX clearance, which adjusted for age, sex, race, and treatment arm (residual from a linear regression model), was included in the dataset. Imputation was performed for each ancestral group using Michigan Imputation Server (Das et al., 2016) and 1KGP Phase 3 was applied as a reference panel. The analysis of variance (ANOVA) and *t*-test were used to test the association between candidate variants and MTX clearance.

### 2.8 Statistical analyses

The Wilcoxon rank-sum test, *t*-test, MLR and LMM were applied to analyze numerical variables, and FET was used for categorical variables. All statistical analyses were performed using R software version 3.6.1.

## 3 Results

### 3.1 Patient characteristics and HD-MTX-induced nephrotoxicity

The patient characteristics are summarized in Table 1. The clinical data of 341 HD-MTX infusions were collected from 51 patients. This study included 28 male (55%) and 23 female (45%). The mean age in total infusions was 9.8 (1.4–17.3) years. Patients received a median of eight HD-MTX (range, 1–8) infusions. Significant differences were found in MTX level at 24, 48, 72, 96, 120, and 144 h, max Cr, min eGFR, delayed MTX clearance percentage, and CTCAE acute kidney injury (AKI) percentage between the total and 51 infusions showing max fold Cr. The baseline eGFR level was normal in all patients, although a statistically significant difference was found between the total and 51 infusions with max fold Cr. Delayed clearance occurred in 80/341 (23.5%) infusions, and 18 patients experienced delayed MTX clearance during the first HD-MTX infusion (Table 1; Supplementary Table S2).

In 51 infusions, the mean MTX concentrations at 24, 48, 72, 96, 120, 144, and 168 h of infusion were 6.36, 1.9, 0.77, 0.59, 0.54, 0.73, and 0.35 mmol/L, respectively. Grade 2–4 AKI developed in 13 (25.5%) of the 51 infusions. One patient (2.0%) experienced grade 4 toxicity and required dialysis. No grade 5 toxicity occurred. Delayed clearance occurred in 25 (49%) of the 51 infusions, showing a maximum-fold increase in Cr.

### 3.2 Correlation of serum MTX levels with toxicity markers

A similar trend was observed in 341 and 51 infusions with the max fold Cr among all toxicity markers (Table 2; Supplementary Table S3). In both total and infusions showing max fold Cr, the 24-h MTX level showed a statistically significant positive correlation (*p*-value<0.01) with the Cr level in nearly all time points. In particular, high correlation coefficients (>0.5) were observed between Cr levels and the MTX level, except for 168 h of infusions showing a max fold increase in Cr. By contrast, other toxicity markers such as ALT, AST, and TB rarely showed significant correlations with the 24-h MTX level.

**TABLE 2:** Correlation of serum MTX levels at 24h with serum creatinine levels.

Measurement	Total infusions		Infusions showing max fold Cr	
	Coefficient	*p*-value	Coefficient	*p*-value
Creatinine				
at 24h	0.20	0.0003	0.57	<0.0001
at 48h	0.38	<0.0001	0.65	<0.0001
at 72h	0.33	<0.0001	0.52	0.0001
at 96h	0.43	<0.0001	0.60	<0.0001
at 120h	0.39	<0.0001	0.50	0.0094
at 144h	0.14	0.1907	0.64	0.0024
at 168h	0.38	0.0003	0.31	0.1699

Coefficient means Pearson’s correlation coefficient.

### 3.3 Variants related to delayed MTX clearance

**FIGURE 2:**
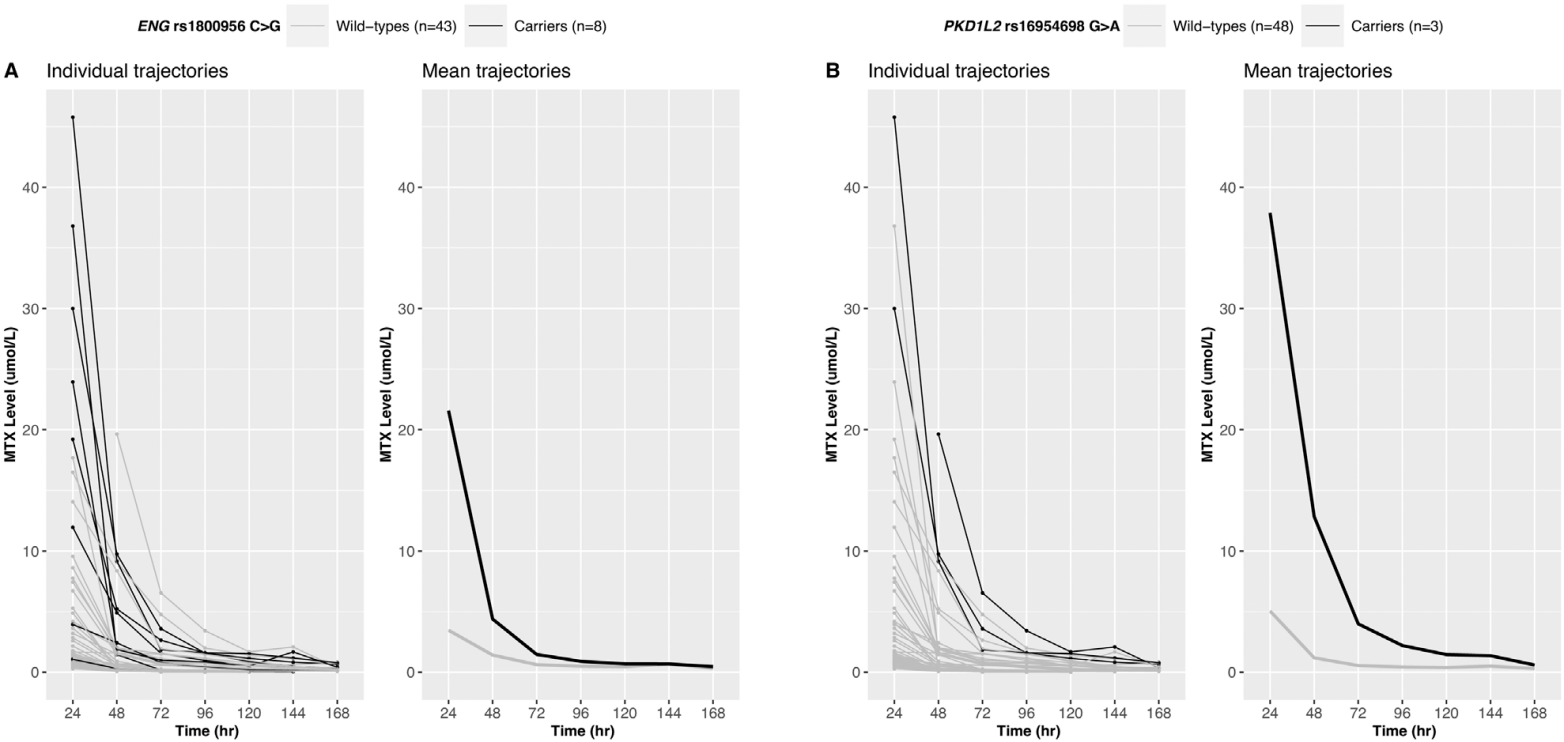
Longitudinal trajectories of serum MTX levels. MTX level changes over time for 51 Korean patients are shown for *ENG* rs1800956 **(A)** and *PKD1L2* rs16954698 **(B)**. Patients carrying the variants exhibited a slower decline in serum MTX levels.

Twelve variants derived from the longitudinal analysis were statistically significant and six variants showed significant associations with the 24-h MTX level (Bonferroni-corrected *p*-value<0.01) (Supplementary Table S4). The intersection of the variants obtained from these two analyses was defined as a candidate variant. The trajectories of MTX levels and candidate variants are shown in Figure 2; Supplementary Figure S1.

To test the binary outcome (case–control), additional analyses of these candidate variants were performed using the FET (Table 3). The case group was defined as patients with delayed MTX clearance. The rs1800956 in *ENG* showed statistical significance in the binary analysis [relative risk: 2.53 (95% CI: 175, 3.66), nominal *p*-value = 0.0017]. Only rs1800956 in *ENG* showed significance in all three statistical tests.

**TABLE 3:** Candidate variants with a statistically significant result in analyses.

Gene	rsID	Allele^†^	AA	P^*^	Case^‡^				Control				FET	
					WT	HET	HOM	AF	WT	HET	HOM	AF	P^**^	RR (95% CI)
*CNTN2*	rs2229866	C>T	P/L	<0.0001	2	2	21	0.88	0	8	18	0.85	0.24	0.47 (0.35, 0.63)
*MTMR9*	rs200687372	A>G	I/V	<0.0001	23	2	0	0.04	26	0	0	0	0.24	2.13 (1.58, 2.87)
*POLI*	rs777260512	T>C	S/P	<0.0001	23	2	0	0.04	26	0	0	0	0.24	2.13 (1.58, 2.87)
*PKD1L2*	rs16954698	G>A	T/M	<0.0001	22	3	0	0.06	26	0	0	0	0.11	2.18 (1.60, 2.97)
*NSMCE1*	rs117765468	A>C	S/R	0.0001	23	2	0	0.04	26	0	0	0	0.24	2.13 (1.58, 2.87)
*ENG*	rs1800956	C>G	D/H	0.0017	17	8	0	0.16	26	0	0	0	<0.01	2.53 (1.75, 3.66)

AA, amino acid changes by *in silico* prediction; WT, wild-types; HET, heterozygotes; HOM, homozygotes; AF, allele frequency; FET, Fisher’s Exact Test; RR, relative risk; CI, confidence interval.

^†^Reference allele > alternative allele.

*Bonferroni-corrected *p*-value derived from longitudinal analysis using linear mixed model.

^‡^Case (MTX, delayed clearance) was defined as serum MTX, level (μmol/L) at 24 h ≥ 15 or 48 h ≥ 1.5 or 72 h ≥ 0.15 or 168 h ≥ 0.1, otherwise defined as control.

**Nominal *p*-value.

### 3.4 Association of ENG rs1800956 and PKD1L2 rs16954698 with toxicity markers

No significant differences were found in baseline clinical indicators such as sex, age, and baseline toxicity markers according to *ENG* rs1800956 and *PKD1L2* rs16954698 (Table 4; Supplementary Table S5). All eight patients with *ENG* rs1800956 exhibited delayed MTX clearance. When reviewing the 341 infusions data, five of the eight patients with *ENG* rs1800956 experienced delayed clearance at the very first HD-MTX infusion (median, 1, range 1–4). In one patient, HD-MTX infusion was permanently discontinued after the first HD-MTX infusion. In six out of seven patients, delayed clearance also occurred in infusions other than the representative infusions (Supplementary Table S2). *ENG* rs1800956 was significantly associated with Cr and eGFR after HD-MTX injection but was not associated with liver function indicators such as ALT, AST, and TB. Heterozygotes of this variant had higher mean Cr (wild type, 0.60; heterozygote, 0.94; *p*-value = 0.0267) and lower mean eGFR (wild type, 105; heterozygote, 75; *p*-value = 0.0475) values than wild types after HD-MTX infusion. In addition, the ratio of AKI severity showed an increasing trend in heterozygotes compared with wild types. Patients with grade ≥1 AKI appeared at a higher rate in heterozygotes (87.5%) than in the wild types (46.5%), although this difference was not statistically significant (*p*-value = 0.0528).

**TABLE 4:** Comparison of the clinical information by genotypes of *ENG* rs1800956.

Characteristics	WT (n = 43)		HET (n = 8)		P *
	Mean ± SD	N	Mean ± SD	N	
Sex (Male, %)		24 (56%)		4 (50%)	1
Age (year)	9.61 ± 4.63	43	10.31 ± 4.18	8	0.5865
Height (cm)	135.28 ± 26.30	43	139.62 ± 28.05	8	0.6976
Weight (kg)	34.80 ± 16.22	43	38.51 ± 18.93	8	0.5954
BSA (m^2^)	1.13 ± 0.37	43	1.21 ± 0.42	8	0.5865
BMI (kg/m^2^)	17.82 ± 2.94	43	18.58 ± 2.96	8	0.2824
MTX dosage (mg)	5560.40 ± 1809.16	43	5913.75 ± 1925.39	8	0.5688
Dosing cycle^†^	3.0 (1–8)	43	2.5 (1–7)	8	0.8015
Baseline Cr (mg/dL)	0.36 ± 0.13	43	0.46 ± 0.21	8	0.1861
Baseline eGFR	164.71 ± 37.32	43	139.45 ± 46.51	8	0.0604
Baseline BUN (mg/dL)	8.59 ± 3.06	42	7.50 ± 1.62	8	0.3798
Baseline ALT (IU/L)	65.00 ± 56.78	42	58.88 ± 53.88	8	0.6913
Baseline AST (IU/L)	42.38 ± 28.5	42	44.25 ± 33.80	8	0.7507
Baseline TB (mg/dL)	0.54 ± 0.27	42	0.54 ± 0.14	8	0.7164
max Cr (mg/dL)	0.60 ± 0.32	43	0.94 ± 0.41	8	0.0267
min eGFR (mL/min/1.73 m^2^)	105.56 ± 29.33	43	75.77 ± 40.04	8	0.0475
max BUN (mg/dL)	11.93 ± 14.61	43	11.49 ± 5.14	8	0.2924
max ALT (IU/L)	197.74 ± 169.71	43	313.88 ± 534.66	8	0.9175
max AST (IU/L)	177.98 ± 197.34	43	222.75 ± 371.81	8	0.7856
max TB (mg/dL)	1.14 ± 0.51	43	1.50 ± 0.96	8	0.2925
CTCAE AKI					
grade≥2		10 (23.3%)		3 (38.0%)	0.4042
grade≥1		20 (46.5%)		7 (87.5%)	0.0528
MTX delayed clearance^‡^		17 (39.5%)		8 (100%)	0.0017

^†^Represented by median (range).

^‡^MTX, delayed clearance was defined as serum MTX, level (μmol/L) at 24 h ≥ 15 or 48 h ≥ 1.5 or 72 h ≥ 0.15 or 168 h ≥ 0.1.

*Wilcoxon rank sum test for continuous variables and Fisher’s exact test for categorical variables.

Of all three patients with *PKD1L2* rs16954698, two had delayed MTX clearance with first infusion and one with the third infusion. Compared to the wild-types of this variant, heterozygotes showed statistically significantly worse status in all renal function indices such as Cr, eGFR, BUN, and grade ≥2 AKI after HD-MTX administration (Supplementary Table S5).

### 3.5 MTX clearance in the replication cohort

Five out of the six candidate variants were genotyped or imputed in the replication cohort (Supplementary Table S6). Among them, only *PKD1L2* rs16954698 and *CNTN2* rs2229866 possessed both wild-types and heterozygotes or homozygotes, enabling a comparison of MTX clearance with the variant. Other variants were rare and had the highest frequency in East Asians in the 1KGP used as an imputation reference panel, but only 1.7% of patients in the replication cohort were Asians, so the genotypes of the variants were only wild-types or no variant was found.

**FIGURE 3:**
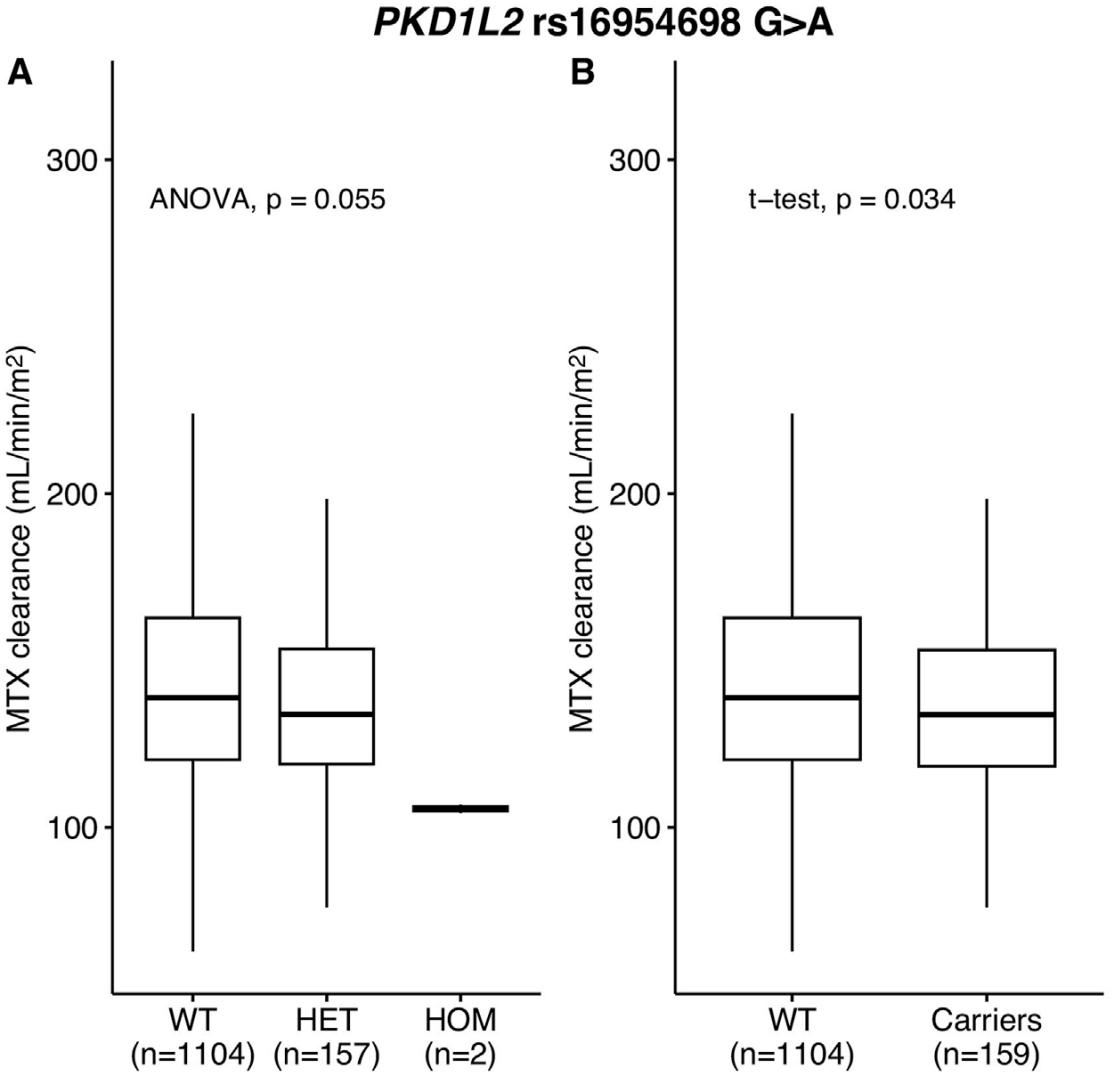
MTX clearance for *PKD1L2* rs16954698 in replication cohort. MTX clearance tends to decrease depending on the A allele of rs16954698 **(A)**. Patients carrying the variant showed a statistically significant reduction in clearance **(B)**. WT, wild-types; HET, heterozygotes; HOM, homozygotes.

MTX clearance was reduced depending on the genotypes of *PKD1L2* rs16954698 (Figure 3A), and the carriers of the variant has significantly lower MTX clearance than the wild-types (*p*-value = 0.034) (Figure 3B). The mean MTX clearance for carriers was 137.57 ± 30.88 (mean ± SD), and for wild-types, it was 143.22 ± 33.82. These results showed similar trends to those observed in the analyses of 51 Korean patients, where patients with the variant had high MTX levels in the blood (Figure 2B; Supplementary Table S4; Supplementary Table S5). On the other hand, in the case of *Contactin 2* (*CNTN2*) rs2229866, there was no significant difference in MTX clearance between the groups (wild-types, 142.88 ± 32.48; carriers, 142.21 ± 34.10) (Supplementary Figure S2). Additionally, this trend was opposite to results in the analyses of 51 Korean patients (Supplementary Figure S1A; Supplementary Table S4).

## 4 Discussion

We identified variants that potentially contribute to delayed HD-MTX clearance from serum measurements after HD-MTX infusion in pediatric patients with ALL. Delayed clearance of HD-MTX increases the risk of severe nephrotoxicity and consequently induces severe life-threatening complications in some cases (Howard et al., 2016). Our data also showed a strong correlation between the 24-h MTX level and the subsequent Cr levels. The incidence of delayed clearance was significantly higher in infusions showing max fold Cr than in total infusions (49% vs 23.5%; *p* = 0.0002). These findings suggest that the peak levels and delayed MTX clearance are associated with subsequent nephrotoxicity.

In this study, 23.5% of the total infusions (80/341 infusions) corresponded to delayed clearance of HD-MTX. AKI occurred in 12.9% of the infusions (44/341 infusions). Delayed MTX clearance has been described in several studies. In a study of pediatric and adult patients with osteosarcoma in China, the frequency of delayed clearance was reported to be 6.19% and 0.86% when delayed and severely delayed clearances were defined as concentration >5 μmol/L at 24 h and concentration >20 μmol/L at 24 h, respectively (Zhang et al., 2016). Another study reported that 22% of patients with lymphoma or patients with leukemia aged 16–84 years experienced delayed clearance of HD-MTX based on the definition of MTX concentration ≥1 μmol/L 48 h or ≥0.1 μmol/L 72 h after HD-MTX (Narita et al., 2017). The frequency of delayed clearance in our study was higher than that reported in other studies, partially because of the broad criteria used.

MTX-induced renal toxicity is caused by the precipitation of MTX and its metabolites in the renal tubules or by the direct toxic effects of MTX on the renal tubules (Garneau et al., 2015). In a meta-analysis of the toxicity of HD-MTX in osteosarcoma, the incidence of grade ≥ II MTX renal toxicity was reported to be 1.5% (range, 0.0%–12.4%) (Widemann et al., 2004). Amitai et al. reported that 13% of patients with hematological malignancies experienced AKI after HD-MTX (median dose, 1941; range, 743–5442 mg/m^2^) (Amitai et al., 2020). Another retrospective study reported that 38.6% of adult patients with hematological malignancies experience renal toxicity (Wiczer et al., 2016).

In particular, renal disorders can reduce renal function and hinder drug clearance (Garza et al., 2022). Renal clearance of drugs depends on the GFR, renal blood flow (RBF), and tubular function (Richard et al., 2020). MTX toxicity may occur unpredictably even without an underlying patient’s condition or external factors, and genetic polymorphisms, such as transporter-related polymorphisms, may be involved. Therefore, the genetic variants associated with delayed clearance and AKI after HD-MTX infusion must be identified.

In this study, *ENG* rs1800956 and *PKD1L2* rs16954698 were identified as promising biomarkers for predicting delayed MTX clearance. These two variants satisfied at least two of the following criteria: *deleterious* (SIFT), *probably damaging* (PolyPhen-2), and *deleteriousness* (CADD) (Supplementary Table S7). Notably, all criteria were met for *ENG* rs1800956. Since SIFT, PolyPhen-2, and CADD are prediction tools that evaluate scores based on the protein’s structure, sequence conservation, and functional information (Eilbeck et al., 2017), these two variants could have both structural and functional impacts on the proteins they encode.

*Endoglin* (*ENG*) is a transmembrane glycoprotein expressed on endothelial cells that functions as a co-receptor for several ligands of the transforming growth factor beta (TGF-β) family (Rossi et al., 2019). The kidney contains various types of endothelial cells. Renal endothelial function plays a role in maintaining to RBF (Basile and Yoder, 2014) and common types of chronic kidney disease (CKD) are characterized by renal endothelial dysfunction (Jourde-Chiche et al., 2019). Specifically, the glomerular endothelium is highly fenestrated, covered by a rich glycocalyx, and plays a key role in the filtration properties of the glomerular barrier and the maintenance of podocyte structure. Capillary glomerular renal endothelial cells show enriched expression of genes associated with the TGF-β–BMP signaling pathway, including ENG, which is involved in glomerular capillary formation. Overexpression of TGF-β induces proteinuria and glomerulosclerosis (Dumas et al., 2021).

Previous studies have shown that TGF-β plays diverse roles in renal fibrosis and inflammation, and an infusion of TGF-β2 resulted in a gradual decline in renal medullary blood (Spurgeon et al., 2005; Gu et al., 2020). Although the most important mechanism of MTX-induced AKI is intratubular crystal precipitation, decreased RBF or urine flow can also affect drug elimination and AKI (Pentel and Benowitz, 1984; Perazella and Luciano, 2015; Garza et al., 2022). Cyclosporine (CSA), an immunosuppressant used in organ and bone marrow transplants as well as inflammatory conditions, stimulates renal and systemic productions of TGF-β, and experimental and clinical studies have suggested that TGF-β overexpression may be an important factor in the development of CSA nephrotoxicity (Burdmann et al., 2003).

Moreover, *ENG* protein is highly expressed in kidney (HPA, 2015), and its vascular expression is elevated in animal models of renal injury, potentially contributing to disease severity by promoting endothelial cell activation and inflammation (Bus et al., 2018). *ENG* is upregulated in various CKD, and interstitial *ENG* expression is correlated with eGFR (Gerrits et al., 2022). In addition, ENG was downregulated by MTX in monocyte-derived human macrophages (Rios et al., 2023). These imply the involvement of ENG in MTX elimination and nephrotoxicity. In our study, *ENG* rs1800956 also showed negative (−0.09) and positive (17.79) coefficients in association with MTX levels and 24 h MTX levels, respectively (Supplementary Table S4). This suggests that heterozygotes for this variant have a slower decline in blood MTX concentration. The allele frequency of *ENG* rs1800956 was higher in Koreans and East Asians than in Europeans with gnomAD (Korea 9.0%, East Asia 10.9%, and Europe 0.1%). The high allele frequency in Koreans might have contributed to the identification of variants as significant in our study.

The *polycystic kidney disease 1-like 2* (*PKD1L2*) gene encodes a polycystin-1-like subfamily, and the protein has ion channel segments that are highly conserved among polycystin-2 family members, suggesting that they possess cation channel functions (Li et al., 2003; Yuasa et al., 2004). According to the Gene Ontology (GO) for *PKD1L2*, the GO classes included calcium ion (Ca^2+^) transmembrane transport and calcium channel activity (AmiGO, 2017). Ca^2+^ is a critical signaling molecule in kidney development, all kidney cellular functions, and emergence of kidney diseases (Cao et al., 2006). The activation of Ca^2+^ channels regulates renal function by controlling the contraction of the renal vasculature, and Ca^2+^ channel blockers significantly increase the RBF and GFR significantly (Cao et al., 2006). Besides, *PKD1L2* was associated with resistance and sensitivity to MTX in the breast cancer and gastric cancer (Udagawa et al., 2020). These studies have indicated that the functions of *PKD1L2* are not only relevant to RBF and drug elimination, but also to be related to MTX.

The *polymerase iota* (*POLI*, Polι), polymerases eta (Polη), kappa (Polκ), and Rev1 belong to the Y-family of polymerases (McIntyre, 2020). The activities of Polι and other noncanonical human polymerases also appear responsible for the toxicity of nucleoside analogs in human immunodeficiency virus and hepatitis B virus (McIntyre, 2020). In addition, a deficiency of Polι in human cell lines was found to result in an increased sensitivity to oxidative damage, and this evidence suggests a role for pol ι in the cellular response to DNA damage induced by oxidative stress (Parsons et al., 2013). Based on these findings, *POLI* may be involved in adverse drug reactions as a drug-related gene.

The hypoxic microenvironment, which downregulates calcium transporter proteins and inhibits apoptotic cell signaling, is considered a contributing factor that promotes DNA instability in prostate cancer cells by upregulating *NSE1 Homolog, SMC5-SMC6 Complex Component* (*NSMCE1*)-related E3 ubiquitin ligases and inhibiting efficient DNA repair (Luo et al., 2020). *NSMCE1* is upregulated in hypoxic environments; however, evidence linking this to drug-induced nephrotoxicity is lacking.

Human *myotubularin-related proteins* (*MTMRs*) *six* and *nine* belong to the myotubularin family (Clague and Lorenzo, 2005). *MTMR6* inhibits the activity of calcium-activated potassium channels (type KCa3.1) and is localized in the plasma membrane (Srivastava et al., 2005; Choudhury et al., 2006; Srivastava et al., 2006). A high *MTMR6* expression level has been observed in B-cell lymphoid leukemia cells with increased resistance to irradiation-induced apoptosis, implicating *MTMR6* as a regulator of apoptosis (Vallat et al., 2003). According to a previous study (Zou et al., 2009), *MTMR9* influences *MTMR6* function at multiple levels, such as by increasing its stability, co-localization, and co-expression with MTMR6. Furthermore, the co-expression of *MTMR6* and *MTMR9* decreased etoposide-induced apoptosis (Zou et al., 2009). *MTMR6* and *MTMR9* may influence the response to chemotherapeutic agents. However, the frequency of some candidate variants was very rare, so replication analysis could not be performed. Therefore, additional analysis is needed on large scale data for these.

*ABCG2* rs2231142, *MTHFD1* rs2236225, *MTHFR* rs1801133, *SLCO1B1* rs2306283 and rs4149056, and *SLC19A1* rs1051266, which were found to be significant in previous studies, were not statistically significant in our study (Cheng et al., 2021; Schulte et al., 2021; Taylor et al., 2021). Other variants in *ABCB1*, *SLCO1B1*, and *ABCC2* did not appear to be associated with delayed MTX clearance in our analysis because they were located in introns, were synonymous variants, or were very rare in East Asians.

If MTX toxicity occurs, an immediate increase in leucovorin dose may be helpful (Ackland and Schilsky, 1987). In severe cases, MTX may be removed through hemodialysis. Recombinant bacterial enzyme glucarpidase has been approved by the US Food and Drug Administration for use in patients with delayed MTX clearance. Glucarpidase converts MTX into its inactive metabolites and provides a nonrenal pathway for MTX elimination. Recently, a clinical decision support tool for predicting delayed MTX clearance was introduced (Taylor et al., 2020). However, delayed clearance was not predicted when MTX was administered. Furthermore, glucarpidase are difficult to use in many countries. Pharmacogenetic findings can help predict MTX nephrotoxicity in advance and allow the use of glucarpidase or other alternative chemotherapeutic agents instead of MTX in patients with variants related to delayed clearance.

This study has some limitations. First, because the variants were relatively frequent in Asians, some were not detected in replication cohorts with few Asians and could not be validated. Therefore, further analysis is required with large-scale datasets involving diverse ethnic backgrounds. Second, future research should be conducted using multi-omics data, such as transcriptomic and epigenomic data, to achieve an integrated understanding. Third, experimental work is needed to how the variants or genes affect MTX levels and toxicities. Due to the low variant frequencies and small sample size in our cohort, identifying interactions between candidate variants was challenging. It is also possible that MTX clearance may be delayed by a related gene variant that is fully linked to the *ENG* rs1800956. Further studies with larger cohorts could help to more clearly elucidate the combined effects of multiple variants, but our study offers a meaningful contribution to understanding individual genetic variability in this context.

## 5 Conclusion

To the best of our knowledge, this is the first study to identify the variants associated with MTX nephrotoxicity in Korean pediatric patients with ALL. In conclusion, *ENG* rs1800956 and *PKD1L2* rs16954698 were the most significantly associated with delayed MTX clearance, and other genetic variants were also found. These findings will help us understand HD-MTX-induced nephrotoxicity and reduce adverse reactions caused by treatment modification.

## Data Availability

The original contributions presented in the study are publicly available. This data can be found here: dbGaP repository, accession number phs000637.v1.p1.
