# Supplementary material for: Novel genomic variants influencing methotrexate delayed clearance in pediatric patients with acute lymphoblastic leukemia
Source: Front Pharmacol. 2024 Nov 14;15:1480657. doi: 10.3389/fphar.2024.1480657 (PMC11603417; doi:10.3389/fphar.2024.1480657)
Supplement: Supplementary file 1 [file DataSheet1.docx]

Supplementary Material

| **Supplementary Table S1. Comparison of toxicity outcomes between total 341 infusions and 51 infusions showing max fold Cr.** | | | | | | |
| --- | --- | --- | --- | --- | --- | --- |
| **Characteristics** | **Total infusions**  **(n=341)** | |  | **Infusions showing max fold Cr**  **(n=51)** | | **P** |
|  | **Mean±SD** | **N** |  | **Mean±SD** | **N** |  |
| Baseline BUN (mg/dL) | 9.10±3.09 | 335 |  | 8.42±2.89 | 50 | 0.0967 |
| Baseline ALT (IU/L) | 57.39±54.67 | 336 |  | 64.02±55.84 | 50 | 0.4811 |
| Baseline AST (IU/L) | 37.20±27.45 | 336 |  | 42.68±29.04 | 50 | 0.2319 |
| Baseline TB (mg/dL) | 0.52±0.25 | 336 |  | 0.54±0.26 | 50 | 0.6453 |
| max BUN (mg/dL) | 9.81±6.66 | 339 |  | 11.86±13.53 | 51 | 0.5004 |
| max ALT (IU/L) | 214.26±230.94 | 341 |  | 215.96±256.97 | 51 | 0.7910 |
| max AST (IU/L) | 163.41±208.70 | 341 |  | 185±228.77 | 51 | 0.6423 |
| max TB (mg/dL) | 1.08±0.51 | 341 |  | 1.19±0.610 | 51 | 0.1999 |
| BUN, blood urea nitrogen; ALT, alanine transaminase; AST, aspartate transaminase; TB, total bilirubin. | | | | | | |

| **Supplementary Table S2. Infusions with delayed clearance and *ENG* rs1800956 and *PKD1L2* rs16954698**. | | | |
| --- | --- | --- | --- |
| **Patient** | **Dosing cycle** | ***ENG* rs1800956** | ***PKD1L2* rs16954698** |
| P01 | 1 | Heterozygotes | Wild-types |
| P02 | 1 | Heterozygotes | Wild-types |
| P02 | 3 | Heterozygotes | Wild-types |
| P02 | 6 | Heterozygotes | Wild-types |
| P04 | 1 | Wild-types | Wild-types |
| P08 | 1 | Wild-types | Wild-types |
| P09 | 3 | Wild-types | Wild-types |
| P09 | 5 | Wild-types | Wild-types |
| P09 | 6 | Wild-types | Wild-types |
| P09 | 7 | Wild-types | Wild-types |
| P09 | 8 | Wild-types | Wild-types |
| P11 | 3 | Heterozygotes | Wild-types |
| P11 | 4 | Heterozygotes | Wild-types |
| P12 | 1 | Wild-types | Wild-types |
| P12 | 2 | Wild-types | Wild-types |
| P12 | 4 | Wild-types | Wild-types |
| P14 | 1 | Wild-types | Wild-types |
| P14 | 2 | Wild-types | Wild-types |
| P14 | 3 | Wild-types | Wild-types |
| P14 | 4 | Wild-types | Wild-types |
| P14 | 5 | Wild-types | Wild-types |
| P14 | 6 | Wild-types | Wild-types |
| P14 | 7 | Wild-types | Wild-types |
| P17 | 4 | Wild-types | Wild-types |
| P20 | 1 | Wild-types | Wild-types |
| P20 | 2 | Wild-types | Wild-types |
| P20 | 5 | Wild-types | Wild-types |
| P20 | 7 | Wild-types | Wild-types |
| P21 | 3 | Wild-types | Wild-types |
| P21 | 6 | Wild-types | Wild-types |
| P21 | 7 | Wild-types | Wild-types |
| P21 | 8 | Wild-types | Wild-types |
| P22 | 1 | Wild-types | Heterozygotes |
| P22 | 2 | Wild-types | Heterozygotes |
| P22 | 3 | Wild-types | Heterozygotes |
| P22 | 4 | Wild-types | Heterozygotes |
| P23 | 3 | Wild-types | Wild-types |
| P23 | 4 | Wild-types | Wild-types |
| P23 | 6 | Wild-types | Wild-types |
| P23 | 7 | Wild-types | Wild-types |
| P24 | 1 | Heterozygotes | Wild-types |
| P24 | 2 | Heterozygotes | Wild-types |
| P26 | 3 | Wild-types | Wild-types |
| P26 | 5 | Wild-types | Wild-types |
| P28 | 1 | Wild-types | Wild-types |
| P28 | 6 | Wild-types | Wild-types |
| P29 | 1 | Wild-types | Wild-types |
| P30 | 1 | Wild-types | Wild-types |
| P32 | 8 | Wild-types | Wild-types |
| P33 | 6 | Wild-types | Wild-types |
| P33 | 7 | Wild-types | Wild-types |
| P34 | 3 | Wild-types | Wild-types |
| P34 | 4 | Wild-types | Wild-types |
| P34 | 5 | Wild-types | Wild-types |
| P34 | 6 | Wild-types | Wild-types |
| P34 | 7 | Wild-types | Wild-types |
| P34 | 8 | Wild-types | Wild-types |
| P36 | 1 | Heterozygotes | Heterozygotes |
| P36 | 2 | Heterozygotes | Heterozygotes |
| P37 | 6 | Wild-types | Wild-types |
| P39 | 1 | Wild-types | Wild-types |
| P39 | 5 | Wild-types | Wild-types |
| P41 | 1 | Heterozygotes | Wild-types |
| P41 | 2 | Heterozygotes | Wild-types |
| P41 | 5 | Heterozygotes | Wild-types |
| P41 | 6 | Heterozygotes | Wild-types |
| P41 | 7 | Heterozygotes | Wild-types |
| P42 | 1 | Wild-types | Wild-types |
| P42 | 5 | Wild-types | Wild-types |
| P43 | 4 | Heterozygotes | Wild-types |
| P44 | 1 | Wild-types | Wild-types |
| P44 | 2 | Wild-types | Wild-types |
| P44 | 3 | Wild-types | Wild-types |
| P44 | 4 | Wild-types | Wild-types |
| P44 | 8 | Wild-types | Wild-types |
| P47 | 6 | Wild-types | Wild-types |
| P49 | 3 | Heterozygotes | Heterozygotes |
| P49 | 7 | Heterozygotes | Heterozygotes |
| P50 | 1 | Wild-types | Wild-types |
| P51 | 6 | Wild-types | Wild-types |

| **Supplementary Table S3. Correlation of serum MTX levels at 24hr with toxicity outcomes.** | | | | | |
| --- | --- | --- | --- | --- | --- |
| **Measurement** | **Total infusions** | |  | **Infusions showing max fold Cr** | |
|  | **Coefficient** | **P-value** |  | **Coefficient** | **P-value** |
| Blood urea nitrogen (BUN) |  |  |  |  |  |
| at 24hr | 0.13 | 0.0192 |  | 0.10 | 0.5155 |
| at 48hr | 0.12 | 0.0311 |  | 0.10 | 0.5028 |
| at 72hr | -0.001 | 0.9866 |  | 0.10 | 0.4861 |
| at 96hr | -0.001 | 0.9840 |  | 0.11 | 0.5126 |
| at 120hr | 0.06 | 0.5230 |  | 0.20 | 0.3189 |
| at 144hr | 0.03 | 0.7676 |  | 0.55 | 0.0264 |
| at 168hr | -0.03 | 0.8205 |  | -0.21 | 0.4178 |
| Alanine aminotransferase (ALT) |  |  |  |  |  |
| at 24hr | 0.04 | 0.4619 |  | -0.10 | 0.4962 |
| at 48hr | 0.005 | 0.9310 |  | -0.21 | 0.1605 |
| at 72hr | -0.07 | 0.2669 |  | -0.22 | 0.1289 |
| at 96hr | -0.07 | 0.2899 |  | -0.11 | 0.5051 |
| at 120hr | 0.16 | 0.0869 |  | 0.56 | 0.0034 |
| at 144hr | 0.28 | 0.0057 |  | 0.53 | 0.0135 |
| at 168hr | 0.32 | 0.0041 |  | 0.75 | 0.0001 |
| Aspartate aminotransferase (AST) |  |  |  |  |  |
| at 24hr | 0.02 | 0.7230 |  | -0.07 | 0.6188 |
| at 48hr | -0.02 | 0.7850 |  | -0.15 | 0.3231 |
| at 72hr | -0.06 | 0.3569 |  | -0.15 | 0.3097 |
| at 96hr | -0.03 | 0.6562 |  | 0.01 | 0.9754 |
| at 120hr | 0.25 | 0.0071 |  | 0.48 | 0.0110 |
| at 144hr | 0.33 | 0.001 |  | 0.48 | 0.0306 |
| at 168hr | 0.27 | 0.0143 |  | 0.65 | 0.0009 |
| Total Bilirubin (TB) |  |  |  |  |  |
| at 24hr | 0.15 | 0.0053 |  | 0.36 | 0.0115 |
| at 48hr | 0.14 | 0.0099 |  | 0.31 | 0.0277 |
| at 72hr | -0.06 | 0.3296 |  | -0.12 | 0.4025 |
| at 96hr | -0.09 | 0.2071 |  | -0.16 | 0.3581 |
| at 120hr | -0.02 | 0.8223 |  | -0.22 | 0.2963 |
| at 144hr | 0.06 | 0.5636 |  | -0.01 | 0.9815 |
| at 168hr | 0.24 | 0.0307 |  | 0.36 | 0.1012 |
| Coefficient means Pearson's correlation coefficient. | | | | | |

| **Supplementary Table S4. Significant variants from longitudinal analysis or analysis with a continuous outcome.** | | | | | | |
| --- | --- | --- | --- | --- | --- | --- |
| **Gene** | **rsID** | **Longitudinal analysis**  **(MTX levels)** | |  | **Analysis with a continuous outcome**  **(24 h MTX level)** | |
|  |  | **Coefficient (95% CI)** | **P** |  | **Coefficient (95% CI)** | **P** |
| *CNTN2* | rs2229866 | 0.20 (0.14,0.25) | <0.0001 |  | -37.57 (-47.19, -27.95) | <0.0001 |
| *MTMR9* | rs200687372 | -0.20 (-0.25,-0.14) | <0.0001 |  | 37.57 (27.95, 47.19) | <0.0001 |
| *POLI* | rs777260512 | -0.20 (-0.25,-0.14) | <0.0001 |  | 37.57 (27.95, 47.19) | <0.0001 |
| *PKD1L2* | rs16954698 | -0.14 (-0.19,-0.1) | <0.0001 |  | 34.92 (23.95, 45.88) | 0.0057 |
| *NSMCE1* | rs117765468 | -0.15 (-0.2,-0.11) | 0.0001 |  | 34.92 (23.95, 45.88) | 0.0057 |
| *ENG* | rs1800956 | -0.09 (-0.12,-0.06) | 0.0016 |  | 17.79 (12.26, 23.32) | 0.0046 |
| *C3orf17* | rs2291465 | -0.16 (-0.21,-0.11) | 0.0004 |  | 24.06 (14.24, 33.88) | 0.7384 |
| *SLC18B1* | rs41286192 | -0.16 (-0.21,-0.1) | 0.0014 |  | 23.51 (13.86, 33.16) | 0.8129 |
| *USP44* | rs74762990 | -0.13 (-0.18,-0.09) | 0.0018 |  | 27.53 (18.64, 36.43) | 0.0105 |
| *GPR135* | rs76859844 | -0.13 (-0.17,-0.08) | 0.0023 |  | 23.84 (13.96, 33.72) | 0.936 |
| *NLRP8* | rs75864168 | -0.11 (-0.15,-0.07) | 0.0038 |  | 15.28 (8.07, 22.5) | 1 |
| *GGT6* | rs11657054 | 0.14 (0.09,0.19) | 0.0079 |  | -24.74 (-34.62, -14.85) | 0.5302 |
| P, Bonferroni-corrected p-value; CI, confidence interval. | | | | | | |

| **Supplementary Table S5. Comparison of the clinical information by genotypes of *PKD1L2* rs16954698.** | | | | | | |
| --- | --- | --- | --- | --- | --- | --- |
| **Characteristics** | **WT (n=48)** | |  | **HET (n=3)** | | **P *** |
|  | **Mean±SD** | **N** |  | **Mean±SD** | **N** |  |
| Sex (Male, %) |  | 27 (56%) |  |  | 1 (33%) | 0.5825 |
| Age (year) | 9.95±4.54 | 48 |  | 5.97±2.22 | 3 | 0.1552 |
| Height (cm) | 137.09±26.43 | 48 |  | 117.90±20.27 | 3 | 0.2073 |
| Weight (kg) | 35.93±16.78 | 48 |  | 26.63±9.16 | 3 | 0.4118 |
| BSA (m^2^) | 1.16±0.38 | 48 |  | 0.93±0.24 | 3 | 0.3267 |
| BMI (kg/m^2^) | 17.89±3.01 | 48 |  | 18.75±0.71 | 3 | 0.2539 |
| MTX dosage (mg) | 5684.94±1831.56 | 48 |  | 4510.00±1161.16 | 3 | 0.2797 |
| Dosing cycle ^†^ | 2.5(1-8) | 48 |  | 4.0(1-7) | 3 | 0.6236 |
| Baseline Cr (mg/dL) | 0.38±0.15 | 48 |  | 0.37±0.10 | 3 | 0.8885 |
| Baseline eGFR | 162.42±39.97 | 48 |  | 133.92±16.59 | 3 | 0.1333 |
| Baseline BUN (mg/dL) | 8.41±2.98 | 47 |  | 8.53±0.81 | 3 | 0.7737 |
| Baseline ALT (IU/L) | 66.11±56.67 | 47 |  | 31.33±28.45 | 3 | 0.236 |
| Baseline AST (IU/L) | 42.89±29.78 | 47 |  | 39.33±15.63 | 3 | 0.6238 |
| Baseline TB (mg/dL) | 0.55±0.26 | 47 |  | 0.43±0.25 | 3 | 0.4673 |
| max Cr (mg/dL) | 0.60±0.26 | 48 |  | 1.54±0.44 | 3 | **0.0061** |
| min eGFR (mL/min/1.73 m^2^) | 105.09±28.65 | 48 |  | 33.51±12.07 | 3 | **0.0048** |
| max BUN (mg/dL) | 11.37±13.68 | 48 |  | 19.73±8.92 | 3 | **0.0285** |
| max ALT (IU/L) | 189.75±162.26 | 48 |  | 635.33±867.08 | 3 | 0.5088 |
| max AST (IU/L) | 168.25±189.18 | 48 |  | 453.00±594.04 | 3 | 0.5889 |
| max TB (mg/dL) | 1.17±0.49 | 48 |  | 1.63±1.79 | 3 | 0.5468 |
| CTCAE AKI |  |  |  |  |  |  |
| grade≥2 |  | 10 (21%) |  |  | 3 (100%) | **0.0137** |
| grade≥1 |  | 24 (50%) |  |  | 3 (100%) | 0.2376 |
| MTX delayed clearance ‡ |  | 22 (46%) |  |  | 3 (100%) | 0.1104 |
| † Represented by median(range) | | | | | | |
| ‡ MTX delayed clearance was defined as serum MTX level (μmol/L) at 24hr≥15 or 48hr≥1.5 or 72hr≥0.15 or 168hr≥0.1. | | | | | | |
| * Wilcoxon rank sum test for continuous variables and Fisher's exact test for categorical variables. Values expressed in bold are statistically significant (nominal p-value < 0.05). | | | | | | |

| **Supplementary Table S6. Six candidate variants in the replication cohort.** | | | | | | | | | | |
| --- | --- | --- | --- | --- | --- | --- | --- | --- | --- | --- |
| **Gene** | **rsID** | **Replication cohort** | |  | **1KGP allele frequency** | | | | | |
|  |  | **Calling** | **Genotypes** |  | **Total** | **EAS** | **EUR** | **AMR** | **AFR** | **SAS** |
| *CNTN2* | rs2229866 | Imputed | WT+Carriers |  | 0.5511 | 0.8760 | 0.7372 | 0.3390 | 0.5591 | 0.3230 |
| *PKD1L2* | rs16954698 | Genotyped | WT+Carriers |  | 0.0663 | 0.0486 | 0.0930 | 0.0497 | 0.1225 | 0.0431 |
| *MTMR9* | rs200687372 | Imputed | Only WT |  | 0.0008 | 0.0040 | 0 | 0 | 0 | 0 |
| *NSMCE1* | rs117765468 | Imputed | Only WT |  | 0.0020 | 0.0099 | 0 | 0 | 0 | 0 |
| *ENG* | rs1800956 | Imputed | Only WT |  | 0.0236 | 0.1111 | 0.0020 | 0.0030 | 0.0014 | 0 |
| *POLI* | rs777260512 | NA | NA |  | NA | | | | | |
| WT, wild-types; EAS, East Asian; EUR, European; AMR, American; AFR, African; SAS, South Asian. | | | | | | | | | | |

| **Supplementary Table S7. The variant effect scores of six candidate variants based on prediction tools.** | | | | |
| --- | --- | --- | --- | --- |
| **Gene** | **rsID** | **Prediction tools** | | |
|  |  | **SIFT** | **PolyPhen-2** | **CADD** |
| *CNTN2* | rs2229866 | 0.23 | 0.022 | **23.6** |
| *PKD1L2* | rs16954698 | **0** | 0 | **18.16** |
| *MTMR9* | rs200687372 | 0.25 | 0 | 13.66 |
| *NSMCE1* | rs117765468 | 0.24 | 0.081 | **17.49** |
| *ENG* | rs1800956 | **0** | **0.971** | **20.3** |
| *POLI* | rs777260512 | 0.09 | 0.003 | 3.397 |
| Scores expressed in bold meet the criteria for being *deleterious* (SIFT), *probably damaging* (PolyPhen-2), and *deleteriousness* (CADD). | | | | |

**
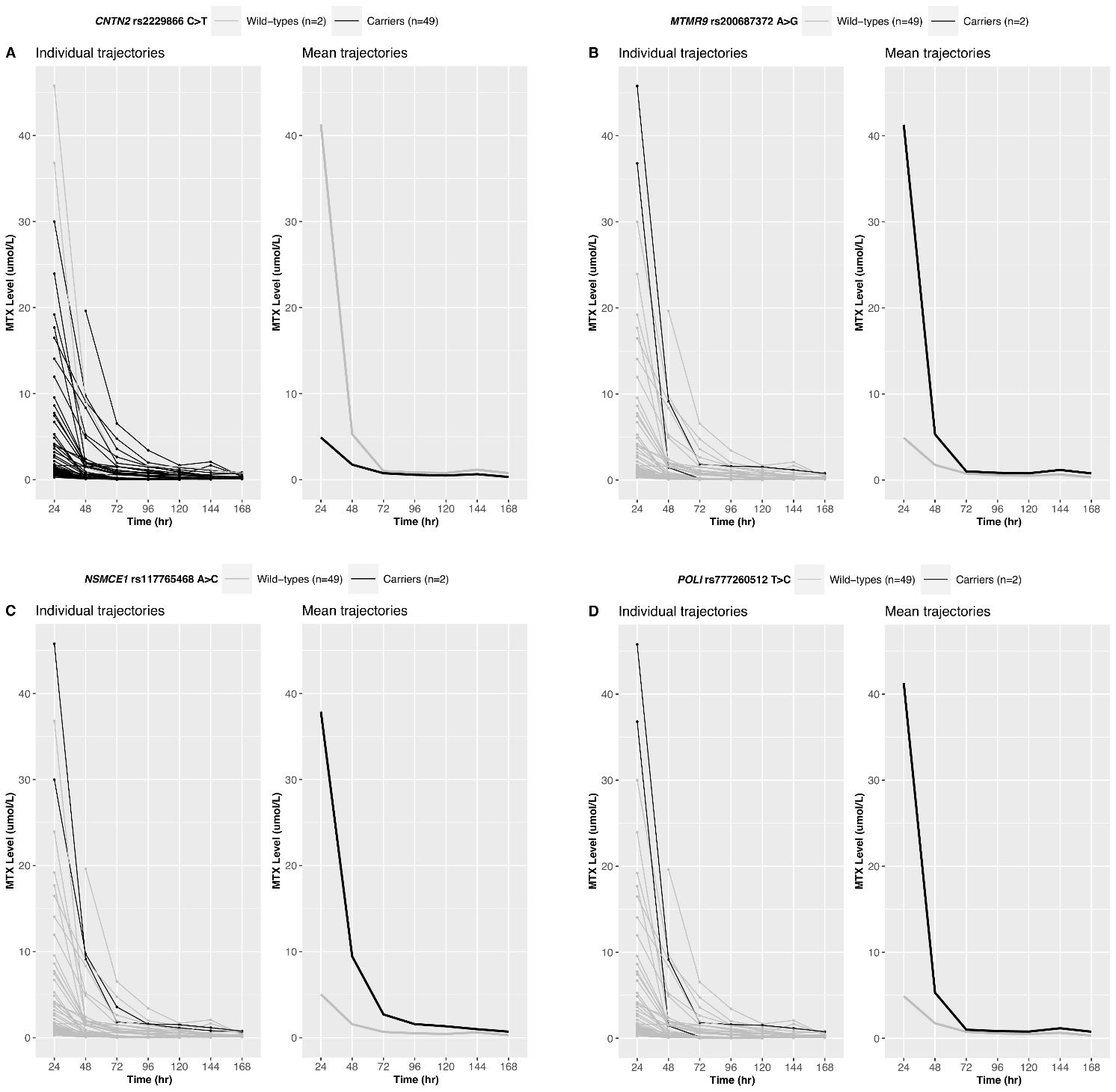
Supplementary Figure S1. Longitudinal trajectories of serum MTX levels for other candidate variants.** Trajectories of MTX level change of 51 Korean patients for *CNTN2* rs2229866 (**A**), *MTMR9* rs200687372 (**B**), *NSMCE1* rs117765468 (**C**), and *POLI* rs777260512 (**D**).

**
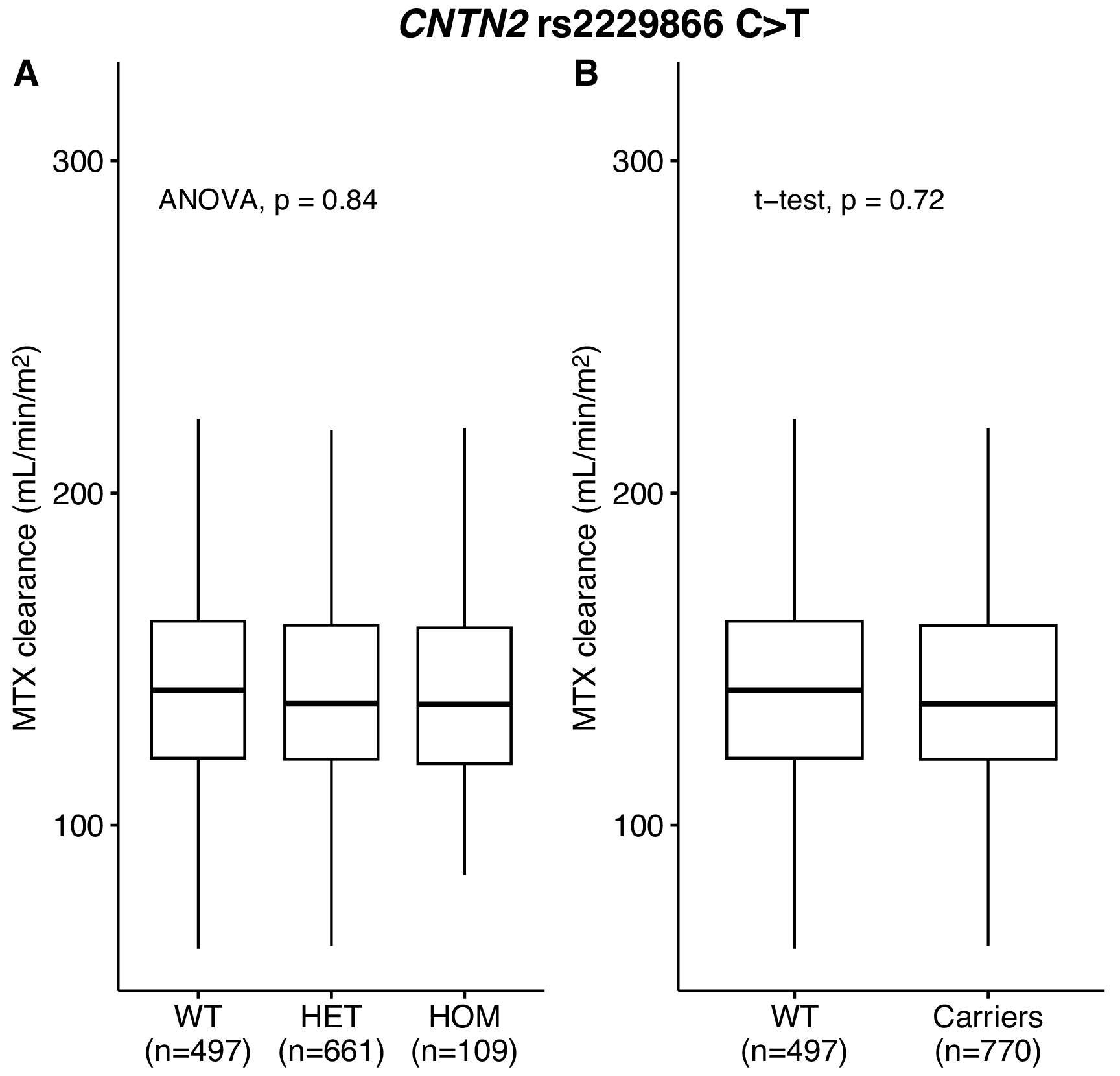
Supplementary Figure S2. MTX clearance for *CNTN2* rs2229866 in the replication cohort.** MTX clearance by genotypes (**A**) and presence or absence of the variant (**B**). WT, wild-types; HET, heterozygotes; HOM, homozygotes.
